# Bioactivity of Fucoidan-Rich Extracts from *Fucus vesiculosus* against Rotavirus and Foodborne Pathogens

**DOI:** 10.3390/md21090478

**Published:** 2023-08-30

**Authors:** Dimitra Graikini, Arturo B. Soro, Saravana P. Sivagnanam, Brijesh K. Tiwari, Lourdes Sánchez

**Affiliations:** 1 Departamento de Producción Animal y Ciencia de los Alimentos, Facultad de Veterinaria, Universidad de Zaragoza, 50013 Zaragoza, Spain; grekinid@gmail.com; 2 Instituto Agroalimentario de Aragón IA2 (UNIZAR-CITA), 50013 Zaragoza, Spain; 3 Foodborne Pathogens Unit, Department of Infectious Diseases in Humans, Sciensano, 1050 Brussels, Belgium; arturo.blazquezsoro@sciensano.be; 4 Teagasc Ashtown Food Research Centre, D15 DY05 Dublin, Ireland; saravana_ps@pukyong.ac.kr (S.P.S.); brijesh.tiwari@teagasc.ie (B.K.T.); 5 Department of Biological Sciences, Munster Technological University, Bishopstown, T12P928 Cork Ireland

**Keywords:** *Fucus vesiculosus*, fucoidans, green extraction, rotavirus, antirotaviral activity, antibacterial assay

## Abstract

Marine algae are sources of bioactive components with defensive properties of great value against microbial infections. This study investigated the bioactivity of extracts from brown algae *Fucus vesiculosus* against rotavirus, the worldwide leading cause of acute gastroenteritis in infants and young children. Moreover, one of the extracts was tested against four foodborne bacteria: *Campylobacter jejuni*, *Escherichia coli*, *Salmonella* Typhimurium, and *Listeria monocytogenes*, and the non-pathogenic: *E. coli* K12. In vitro tests using MA104 cells revealed that both whole algae extracts and crude fucoidan precipitates neutralized rotavirus in a dose-responsive manner. The maximum neutralization activity was observed when the rotavirus was incubated with 100 μg mL^−1^ of the hydrochloric acid-obtained crude fucoidan (91.8%), although crude fucoidan extracted using citric acid also demonstrated high values (89.5%) at the same concentration. Furthermore, molecular weight fractionation of extracts decreased their antirotaviral activity and high molecular weight fractions exhibited higher activity compared to those of lower molecular weight. A seaweed extract with high antirotaviral activity was also found to inhibit the growth of *C. jejuni*, *S*. Typhimurium, and *L. monocytogenes* at a concentration of 0.2 mg mL^−1^. Overall, this study expands the current knowledge regarding the antimicrobial mechanisms of action of extracts from *F. vesiculosus*.

## 1. Introduction

Every year, nearly half a million children under the age of five die of acute infectious gastroenteritis around the world [1]. Among all diarrheic agents, rotaviruses (RV) are the leading etiologic causes of severe diarrhea in neonates and children under five years, accounting for approximately 258 million morbidity cases and 128,000 deaths every year in the world [2,3]. Although vaccination is being successfully used to reduce the severity of diarrhea caused by RV, in most developed countries, it is not yet completely effective in countries of low socio-economic conditions [4,5]. In fact, a systematic review on RV mortality for children under five years found that more than 90% of those with fatal RV infections live in low-income countries [6]. Reasons for ineffective vaccination are mainly associated with environmental factors, such as climate, poverty, malnutrition, genetic factors, water pollution [7], and financial and logistic problems [8]. Therefore, the searching for alternative antirotaviral agents is crucial as it could have several advantages, e.g., lower toxicity, higher effectiveness, broader range of action (also towards new variants), and lower cost, as well as for a better worldwide supply, for example, to the poorest countries [9,10].

At the same time, foodborne bacteria such as *E. coli*, *Salmonella* spp., and *C. jejuni* have also been found to invade and infect the intestinal tract of neonates and children, leading to watery or bloody diarrhea, dysentery, or persistent diarrhea. Moreover, these infections may result in complications such as reactive arthritis, complete Reiter’s syndrome (characterized by arthritis, uveitis, and conjunctivitis), hemolytic–uremic syndrome, and thrombotic thrombocytopenic purpura (caused by Enterohaemorrhagic *E. coli*), as well as Guillain–Barré syndrome (a polyneuropathy caused by *C. jejuni*) [11]. Furthermore, the causative agents of these illnesses are found to contaminate numerous sources, including soil, water, food, air, humans, and processing equipment. The foodborne pathogens most commonly associated with outbreaks worldwide are *Campylobacter* spp., *Salmonella* spp., Shiga toxin-producing *E. coli*, and *L. monocytogenes*. Among these, the latter pathogen has shown the highest number of deaths [12]. In recent decades, efforts have been made to identify and evaluate novel strategies for reducing the prevalence of these foodborne pathogens. One promising intervention to ensure food safety in the agricultural and food sectors is the application of natural antibacterial substances [13].

In this context, the marine ecosystem is the most biodiverse ecosystem in our planet [14]. Marine algae are continuously exposed to several environmental stresses, such as high salinity, high pressure, low temperatures, and lack of nutrients; thereby, they have adapted to these extreme conditions by developing a wide variety of biomolecules [15]. In recent decades, these compounds have attained great interest from the scientific community, as in vivo and in vitro studies have reported that they possess robust antibacterial [16], anticancer [17,18,19], and antioxidant [20] qualities, as well as immunity-boosting effects [21]. With regards to their antiviral activity, several studies have shown that components deriving from marine sources successfully hinder viral infections utilizing diverse mechanisms of action, such as immunostimulatory, anti-inflammatory, antioxidant, and direct antiviral activity [9,22]. Hence, viruses, as a rule, do not acquire resistance to those compounds [23].

The currently identified antiviral and antibacterial constituents derived from seaweed are specific polysaccharides, glycoproteins (i.e., lectins), and polyphenolic compounds (i.e., phlorotannins) [24]. Sulfated polysaccharides, such as carrageenans, ulvans, fucoidans, and alginates, are negatively charged natural complex polymers found mainly in the cell walls of marine algae [25], and are intensively investigated to analyze their beneficial biological activity [26]. Their complexity in constituents, structural conformations, size, and functional groups (i.e., degree of sulfation) significantly influence their antiviral properties [27,28]. Thus, depending on these characteristics, they play an essential role in boosting the host’s defenses by impeding one or more stages of the virus attachment, adsorption, and replication process [29] or improving the host antiviral immune responses to accelerate the process of viral clearance [30]. Furthermore, sulfated polysaccharides possess strong antimicrobial activity against a wide range of bacteria, including both gram-positive and gram-negative strains. They exert their antibacterial effects by disrupting the bacterial cell membrane and interfering with vital cellular processes, ultimately leading to microbial death or growth inhibition [31]. The antimicrobial activity of seaweed extracts from different algae species is currently being explored for application in the agri-food sector. Their use is particularly focused on the prevention and control of foodborne pathogens and the extension of shelf life in food products [32]. The possibility of incorporating seaweed extracts as alternatives to improve food safety is promising, given the substantial amount of data in the scientific literature that supports this statement [31].

The objective of the present study has been to obtain fucoidan-rich extracts from *F. vesiculosus* and to investigate their antiviral and antibacterial potential. Antirotaviral activity was evaluated in vitro on MA104 cells using an indirect immunofluorescence assay. Furthermore, the mechanism of antirotaviral action and the influence of molecular weight fractionation was studied. Additionally, the antibacterial activity of extracts against four different foodborne pathogens, *C. jejuni*, *E. coli*, *S*. Typhimurium, and *L. monocytogenes*, and a non-pathogenic bacterium, *E. coli* K12, was evaluated. This study will contribute to expand the current knowledge on the identification of natural bioactive components as complementary strategies for the treatment of certain diseases of microbial origin.

## 2. Results and Discussion

### 2.1. Extraction of Fucoidan from Seaweed

In this study, the extraction of fucoidan was attempted using conventional methods, by freeze-drying it directly without ethanol precipitation and by subsequent molecular weight cut-off (MWCO) fractionation, using various solvents as extraction media. The MWCO approach of obtaining fucoidan will provide a greener method of production. The fucoidan content was measured based on the fucose content in the extract. The fucoidan content of the extracts and MWCO fractions is shown in Table 1 and Table 2. The results showed that the use of hydrochloric acid (HCl) as a solvent, compared to the use of citric acid (CA), gave a higher amount of fucoidan; this result was observed for the whole extract, crude fucoidan, and MWCO fractions. However, in the MWCO samples the F1 (>300 KDa) showed the highest content of 63.59 ± 1.01 (g fucoidan/100 g extract). In studies performed in other types of algae, fucoidan content has also been determined. Thus, fucoidan extracted from *Ascophyllum nodosum* using a MWCO of 10 kDa showed a content of 562.3 ± 0.08 mg/g of dry seaweed extract [33]. Another report on *Padina boryana* showed a fucoidan content of 1.59 ± 0.16%, expressed in dry weight, in an extract obtained by using a 10 kDa MWCO fractionation [34]. Likewise, as in other studies valuing fucoidans obtained via ultrafiltration or MWCO fractionation, the chemical composition and structure elucidation of the most promising fractions will deserve further study and explanation [35,36]. Similar to these results, Rioux et al. [37] stated significant differences in the content and chemical structure of fucoidan extracted from *Ascophyllum nodosum*, *F. vesiculosus*, and *Saccharina longicruris* using a similar extraction protocol. Earlier reports suggest that alterations in the contents of fucoidan and other elements in macroalgae depend on various aspects, such as the season, species, and location of collection [38,39,40].

### 2.2. Cell Viability

An antimicrobial compound should combine high effectiveness with the lowest cytotoxicity. *F*. *vesiculosus* is one of the 22 species of seaweed that is considered safe to be consumed as a vegetable and a condiment under the European directive (EC 258/97) [41]. This means that the extracts of these algae are not cytotoxic and could be used as a component in pharmaceuticals. Since the extraction method has been reported to drastically affect the composition [42] and, therefore, the properties of the final extract, it was important to investigate the potential cytotoxic effect that the extracts produced in this study might have on the cells. For this, MA104 cells were incubated with algae extracts in several concentrations and the viability was measured using the MTS colorimetric assay.

**Figure 1:**
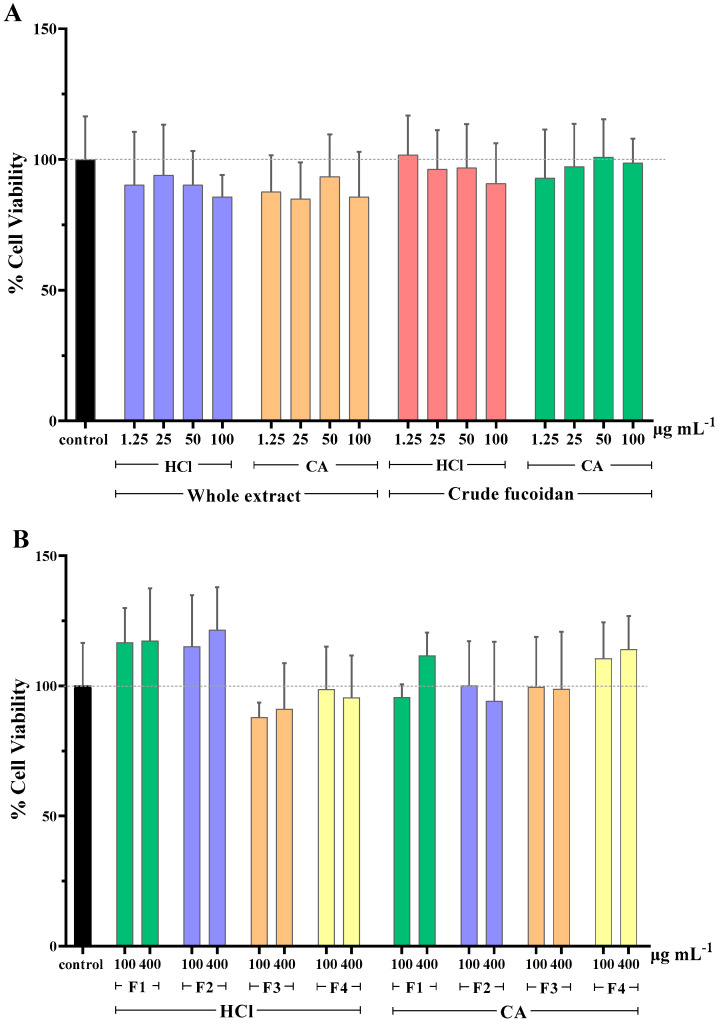
Cell viability (%) of MA104 cells incubated with (**A**) whole extracts and crude fucoidan, and (**B**) MWCO fractions obtained from the whole extracts. F1: Fraction > 300 kDa, F2: Fraction < 300 kDa, F3: Fraction < 100 kDa, F4: Fraction < 10 kDa, HCl: 0.1 M hydrochloric acid, and CA: 1% (*w*/*v*) citric acid solution. Results are shown as mean ± standard deviation of duplicates from two independent experiments (*n* = 4). No significant differences between the cell viability of control and tested samples were found (*p* ≥ 0.05).

Overall, the majority of extracts reduced slightly cell viability compared to the control (Figure 1A). The lowest value of viability was 84.9% and it was observed when cells were treated with the whole CA extract at a concentration of 25 μg mL^−1^. Exceptions were the cells treated with crude HCl fucoidan fraction at 1.25 μg mL^−1^ and crude CA fucoidan fraction at 50 μg mL^−1^, which resulted in values of cell viability of around 101%. However, when comparing the viability values of the treated and untreated cells, no significant differences were found for any of the concentrations tested.

Interestingly, when assessing the cytotoxicity caused by the MWCO fractions of whole extracts, it was observed that those of lower molecular weight (F3 and F4, <100 KDa), independently of the extraction solvent used, produced an increase in cell viability percentages (Figure 1B). This effect was not observed for the extracts of higher molecular weight (F1 and F2, >100 KDa). It is possible that some of the extracted molecules in these samples acted as a nutritional supplement for cell culture, producing an increase in the metabolic activity that the MTS assay measures. Again, neither the MWCO fractionation (F1–4) nor the extraction solvent selection (HCl, CA) significantly affected the cell viability when compared to untreated cells.

In similar studies, fucoidans extracted from various algae sources, screened for their potential antiviral activity, were not found to cause cytotoxicity. A recent study revealed that fucoidan extracted from *Scytosiphon lomentaria* showed anti-HSV-1 and anti-HSV-2 activity with no cytotoxicity up to 1000 μg mL^−1^ (CC50 > 1000 μg mL^−1^) [43]. With regards to anti-HIV activity, α-L-fucan and galactofucan, which are high molecular weight fucoidans isolated from *Saccharina cichorioides* and *S. japonica*, respectively, did not exert cytotoxicity at concentrations up to 100 μg mL^−1^ [44]. Additionally, two lower molecular weight fucoidan fractions of 45 kDa and 30 kDa, isolated from *Sargassum swartzii,* showed no toxicity up to 1000 μg mL^−1^ [45]. When cytotoxicity of fucoidans from the algae *Cladosiphon okamuranus* were compared with those of the well-known antiviral drug Ribavirin, it was found that the former was able to exert higher antiviral activity against Newcastle disease virus while presenting lower cytotoxicity [46].

### 2.3. Antirotaviral Activity

Fucoidan from brown algae has demonstrated a potent effect against both DNA [47,48,49] and RNA viruses [46,50,51,52]. However, there are still challenges associated with the production of extracts rich in bioactive molecules that need to be addressed [42]. In this view, the present study investigated the in vitro antirotaviral capacity of extracts from *F. vesiculosus*, obtained with two different solvents: HCl and CA. We firstly assessed the neutralization activity (direct virucidal effect) of the whole algae extract after alginate separation and, subsequently, the neutralization activity of the crude fucoidan fraction obtained via ethanol precipitation of the whole extract. Additionally, the activity of the crude fucoidan fractions to block cell receptors (protective effect) was investigated to achieve a better understanding of the antirotaviral mechanism.

**Figure 2:**
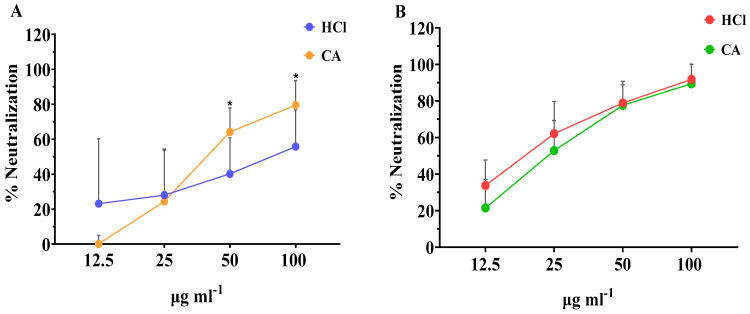
Effect of the concentration of (**A**) whole extracts and (**B**) crude fucoidan extracted with CA or HCl from algae samples on their neutralization activity against bovine RV WC3 infection of MA104 cells. Results are shown as mean ± standard deviation of duplicates from four independent experiments (*n* = 8). Significant differences between the means of same concentrations are indicated with * *p* < 0.05.

As seen in Figure 2A, extracts obtained with both types of solvents were able to neutralize RV in a dose-responsive manner. At low concentration, of 12.5 μg mL^−1^, the neutralization effect of the extracts obtained with CA was lower than that obtained with the HCl extracts. However, when tested in higher concentrations, the CA extract showed significantly higher antirotaviral activity compared with the HCl extract, with neutralization values of 79.6% and 55.8%, respectively. When assessing the neutralization capacity of the crude fucoidan fractions, even higher antirotaviral potential was observed (Figure 2B). These fractions presented neutralization values up to 91.8% for the HCl fraction and 89.5% for the CA fraction at the highest concentration tested. Similar to the whole extracts, the neutralization profile of the crude fucoidan fractions, followed a dosis-effect profile, and no significant differences were observed in any of the neutralization percentages between each concentration tested. Overall, the concentration of all types of algae samples evaluated should be >100 μg mL^−1^ to impede completely the infectivity by RV.

Our results are in accordance with those reported by Mofeed et al. (2022) who reported that extracts from the red seaweeds *Amphiroa anceps* and *Corallina officinalis* exhibited high antiviral effect against simian RV infection, reducing the viral titers by 45.8 and 41.6%, respectively [53]. In this study, extracts were obtained by the Soxhlet method using methanol and hexane as a solvent mixture and the authors attributed the activity to secondary metabolites. Similarly, another report showed that methanol and *n*-hexane extracts obtained from *Spirulina platensis* were significantly active against human RV showing mean inhibition of 56.7 and 66.7%, respectively; however, aqueous extracts failed to show any activity whatsoever [54]. In our study, the neutralization values achieved with either extracts or crude fucoidan obtained with CA were equal and/or greater to those observed with the corresponding HCl samples. These results demonstrate the feasibility of replacing organic solvents by green solvents, such as citric acid, in the extraction processes, and, therefore, being in line with the principles of green chemistry [55,56].

The structure of fucoidan from the genus *Fucus* spp. consists of alternating (1→3)- and (1→4)-linked α-l-fucopyranose residues with sulfate groups occurring at the position C-2 of the 3-linked fucose residues and positions C-2 and -3 of the 4-linked fucose residues [57,58]. It has been reported that the antiviral activity of fucoidans from *F. vesiculosus* is directly related with its degree of sulfonation [59,60]. Sulfate groups are believed to exert antiviral activity in two main pathways, including (a) direct interaction with the positively charged viral surface proteins leading to the neutralization of the virus and (b) blocking virus interaction with the cell receptors via their polyanionic features and, therefore, obstructing the attachment of the viral particle to the host cell [9,26,61]. Recently, in vitro studies in Vero and human MT-4 cells revealed that native and enzymatic modified fucoidans had beneficial effects against HSV-1, HSV-2, ECHO-1, and HIV-1 by inhibiting virus infection in different phases of cell pretreatment, virus pretreatment, simultaneous treatment, and treatment of infected cells [62]. The results of these studies confirmed that the antiviral effect of fucoidans is primarily exerted during the early stages of viral infection, but the exact mechanism of action strongly depends on the type of virus [9,62].

To our knowledge, there are no previous reports regarding the activity of brown algae and, specifically, of *F. vesiculosus* against RV. The outermost layer of this virus is composed of two proteins, VP4 and VP7 [63], which are responsible for the initial interactions of virions with the host cell receptors and virus entry. The viral protein VP4 (VP8 domain) interacts with a sialylated cellular receptor that mediates the initial binding of the infectious particle to the cell surface. In the post-binding phase, both VP4 (VP5 domain) and VP7 interact with several cell surface molecules, including integrins and gangliosides, which seem to act as cellular co-receptors that allow the virus to gain access into the cell [64]. In the in vitro neutralization model used in the present study, pretreatment of virus suspensions with fucoidan-rich fractions, resulted in significantly lower infection levels. Furthermore, pretreatment of the cells with the algae extracts did not show any significant antiviral effect. Therefore, it can be hypothesized that one of the possible mechanisms of the high antirotaviral activity of fucoidans from *F. vesiculosus* is related to the inactivation of RV by the blockage of one or both virus surface proteins VP4 and VP7.

It is important to mention that, apart from fucoidans, it is possible that more molecules with antiviral activity could have been coextracted in this study, especially in the case of the evaluation of the whole extracts. In this regard, lectins are glycoproteins found also in algae, and they have demonstrated antiviral activity against different viruses as they are unique to certain sugars such as mannose, lactose, and other glycans [65]. The carbohydrates recognition domain (CRD) of lectins determines the specificity and avidity of sugar-binding properties [65]. Lectins have the ability to bind reversibly to viral receptors and viral glycoproteins in a non-covalent and highly specific manner resulting in inhibition of infection [66,67]. Moreover, a heterogenous group of polyphenolic compounds called phlorotannins, found in abundance in brown seaweed [68], are considered one of the strongest viral inhibitors derived from algae. Phlorotannins combine several mechanisms of antiviral action, both direct via blockage of viral attachment and replication, and indirect via boosting the host’s immune responses and suppressing pro-inflammatory production pathways caused by the infection [69].

Apart from the degree of sulfonation of the fucoidan, another factor that greatly affects the bioactivity of this molecule is the degree of polymerization [19]. Hence, we sought to investigate how the MWCO fractionation of extracts from *F. vesiculosus* influenced their antirotaviral activity. Therefore, to identify the most active fractions, whole HCl and CA extracts were subjected to ultrafiltration through different pore size cellular membranes and then, fractions were tested for their in vitro neutralization activity.

**Figure 3:**
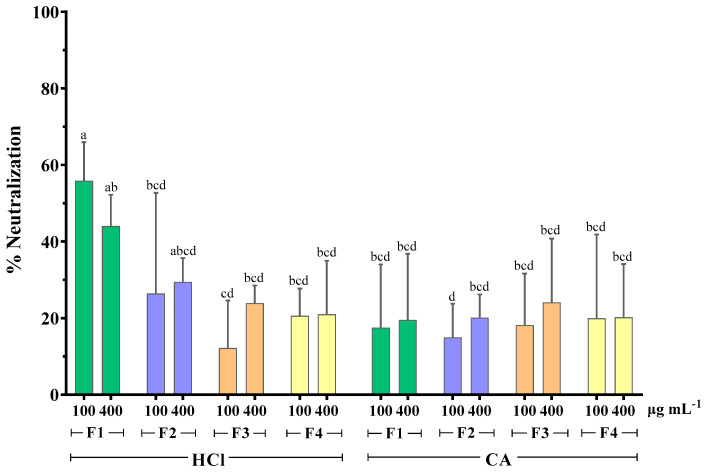
Effect of MWCO fractionation on the neutralizing activity of whole extracts from *F. vesiculosus* against bovine RV WC3 strain infection of MA104 cells. F1: Fraction > 300 kDa, F2: Fraction < 300 kDa, F3: Fraction < 100 kDa, F4: Fraction < 10 kDa, HCl: hydrochloric acid 0.1 M, CA: citric acid solution 1% *w*/*v* of duplicates from three independent experiments (*n* = 6). Superscript letters (a, b, c, and d) express statistical significance for *p* < 0.05 or higher.

As seen in Figure 3, the MWCO fractionation of extracts obtained with HCl as extraction solvent seemed to affect their antirotaviral activity. More specifically, it was shown that the RV neutralization was directly proportional to the molecular weight of the sample tested. The highest neutralization percentage was 55.9% and it was observed when RV was preincubated with the HCl fraction of the highest molecular weight (F1 > 300 kDa). Contrary to this, the extracts obtained with the CA method did not follow this pattern, as all fractions exhibited similar activity, which was considerably low, with neutralization values ranging from 15 to 24.1%. These results indicate that the extraction method influences the antirotaviral activity of the fractions.

It has been previously reported that the antiviral activity of algae polysaccharides increased with their molecular weight. Prokofjeva et al. screened several natural fucoidans from brown algae for their anti-HIV-1 activity and concluded that the fucoidans of high molecular weight were the most active anti-HIV agents, with efficiency against lentiviral transduction demonstrated at low concentrations (0.001–0.05 µg mL^−1^) [44]. High molecular weight fucoidan (~536 kDa) from the brown alga *Kelmanella crassifolia* was shown to bind and block influenza A virus neuraminidase activity, inhibiting the release of viral particles [51]. Krylova et al. (2020) also reported that the native 160 kDa form of fucoidan from *Fucus evanescens* inhibited the replication of HSV-1 and -2 more effectively than its enzymatically modified fraction (50.8 kDa) [62]. However, no differences in the antiviral activity of both fractions were observed for HIV-1 and ECHO-1.

HPLC analysis of fucoidan-rich extracts from *F. vesiculosus* showed that both standard commercial fucoidan and the fucoidan fraction of molecular weight >300 kDa had one marked sharp peak in common in their chromatograms. However, the chromatogram of the whole algae extract revealed an unresolved second peak [36]. The same authors showed that total glycan and protein content was primarily retained in the higher molecular weight fraction (>300 kDa). Considering all the above data, it is possible that MWCO fractionation causes a decrease in the structural fragment’s diversity in the fucoidan molecule, and, consequently, a reduction in the number of potential fucoidan targets on the viral surface, resulting in lower antirotaviral activity.

At the same time, the importance of the lower molecular weight components on the antiviral activity should not be ruled out. In our study there was evidence of mild antirotaviral activity exerted by fractions of molecular weight even lower than 10 kDa (F2, F3, and F4). It has been reported that some low-molecular weight compounds, such as heparin-derived oligosaccharides (degree of polymerization: 10–12), pentosan polysulfates (3 kDa), dextran sulfates (5 kDa), and PI-88 analogs, still possess an antiviral potential [70,71]. An alternative method of rendering low-molecular weight compounds to express antiviral activity may be the formation of a higher order structure, such as helixes, that may recognize complementary regions on target proteins [72].

Finally, apart from the role that the molecular weight of fucoidans plays on the antiviral activity, it has been shown that this property, along with the sulfonation degree, is a determinant for several types of bioactivities, such as antibacterial [73], antitumor [74], anti-angiogenic [75], and immunomodulatory effects [76].

### 2.4. Antibacterial Activity

Few studies have evaluated the antibacterial activity of seaweed extracts towards foodborne pathogens [77,78,79]. The present study examined the antibacterial activity of the seaweed fraction F1 (MWCO of >300 KDa) obtained using the HCl extraction solvent, against four different foodborne pathogens, *C. jejuni*, *E. coli*, *S*. Typhimurium, and *L. monocytogenes*, and a non-pathogenic bacterium, *E. coli* K12. This fraction was chosen based on its previously determined high antiviral activity. Different concentrations (0.2, 0.4, 0.78, 1.56, 3.13, and 6.25 mg mL^−1^) of the extract were assayed with the five aforementioned bacteria to determine the minimum inhibitory concentration (MIC). In Table 3, the MIC findings for the different bacteria are summarized.

Among the tested bacteria, pathogenic *E. coli* and *E. coli* K12 were found to be the less susceptible bacteria to the assayed extract. *E. coli* growth was inhibited at an extract concentration of 6.25 mg mL^−1^, while *E. coli* K12 was inhibited at 3.13 mg mL^−1^. *S*. Typhimurium was able to grow at the lowest tested concentration of 0.2 mg mL^−1^ and showed inhibition at all higher concentrations. Both *C. jejuni and L. monocytogenes* were inhibited at all the tested concentrations, indicating that their MICs were below 0.2 mg mL^−1^. The MIC of the seaweed extract was determined to be 6.25 mg mL^−1^ which effectively inhibited the growth of all the tested bacteria. To the best of the authors’ knowledge, the antibacterial effect of *F. vesiculosus* on foodborne pathogens has not yet been reported in the scientific literature. Hornsey et al. examined the inhibitory effect of the seaweed itself and not on a *F. vesiculosus* extract and did not observe any evidence of antibacterial activity towards *E. coli* and *S. aureus* [80]. In another study, the MIC of 3.125 mg mL^−1^ has been observed for a *F. vesiculosus* extract that inhibited the growth of *S. aureus* [81]. The antimicrobial effect of *F. vesiculosus* has been attributed to fucoidan. Thus, a fucoidan concentration of 250 µg mL^−1^ inhibited the growth of oral gram-positive bacteria and their capacity to form biofilms [82]. The present study observed how a *F. vesiculosus* extract with a high concentration of fucoidan (63.59 g fucoidan/100 g extract) could suppress the growth of *E. coli*, *C. jejuni, S*. Typhimurium, and *L. monocytogenes*. Despite these findings, further analysis and additional information about the specific antibacterial properties of the seaweed extract would be necessary for a more comprehensive understanding.

## 3. Materials and Methods

### 3.1. Chemicals and Samples

l-(−)-Fucose, sulfuric acid (95–97%), L- cysteine hydrochloride, food grade citric acid, and hydrochloric acid were purchased from SIGMA (Sigma-Aldrich, Saint Louis, MO, USA). Minimal essential medium (MEM), heat-inactivated fetal bovine serum (FBS), l-glutamine, antibiotics (penicillin-streptomycin), amphotericin B, and trypsin-EDTA were obtained from Gibco (Life Technologies Corporation, Paisley, UK). Specific antiserum against bovine RV obtained in lamb was kindly donated by Dr Snodgrass of Moredun Research Institute (Penicuik, UK). Donkey anti-sheep immunoglobulin antiserum conjugated with fluorescein isothiocyanate (FITC), gelatin from porcine skin, and trypsin from porcine pancreas used for RV activation were purchased from Sigma-Aldrich (Saint Louis, MO, USA). Mueller Hinton broth (MHB), trypticase soy agar (TSA), maximum recovery diluent (MRD), and Mueller Hinton agar (MHA) was purchased from Oxoid (Basingstoke, UK). Gentamicin was obtained from Sigma-Aldrich (Wicklow, Ireland).

Bovine RV strain WC3 (VR-2102) and the rhesus monkey epithelial cell line MA104 (ATTC CRL-2378) were purchased from the American Type Culture Collection (ATCC, Manassas, VA, USA). *E. coli* K12, *C. jejuni* NCTC 11168, *E. coli* ATCC 25922, *S. enterica* Typhimurium ATTC 14028, and *L. monocytogenes* Scott A were selected for the antibacterial method from the microbiological culture collection at the Teagasc Food Research Centre, Ashtown (Dublin, Ireland).

### 3.2. Seaweed Samples

*F. vesiculosus* were harvested in March 2019 in Galway Bay by BEOBIO (Leitir Mór, Connemara, Co., Galway, Ireland). The seaweed was washed and cleaned thoroughly to remove dirt and other foreign materials and these samples were oven-dried (50–60 °C, 2 days) and subsequently milled to 1 mm particle size using a hammer mill.

### 3.3. Extraction of Fucoidan from Seaweed

Seaweeds (*F. vesiculosus*) were used in a large-scale extraction process with a mixing ratio of 500 g/5 L using either 0.1 M HCl or 1% CA for 2 h at 80 °C. This process was carried out using a Buchi Rotavapor R-220 (Mason Technologies, Dublin, Ireland). Next, extracts were centrifuged for 30 min at 8000 rpm with 4 °C using a Sorvall Lynx 6000 centrifuge (Fischer Scientific Ireland, Dublin, Ireland). After the filtration process, the supernatant was added with 1% CaCl_2_ in an equal volume to that of the supernatant. Alginate was separated after this process by centrifugation at 8000 rpm for 30 min at 4 °C, and the supernatant liquid was divided into fractions of 2 L each for three different processes, such as freeze-drying, ethanol precipitation of crude fucoidan, and separation of fractions with different molecular weight by ultrafiltration.

One fraction of the supernatant obtained as previously described was freeze-dried and, thereof, called “whole extract”. Another fraction of the supernatant was added with (1:3 *w*/*v*) absolute alcohol and it was kept overnight. Then, it was centrifuged at 8000 rpm, 30 min and 4 °C. This fraction containing the fucoidan was freeze-dried and, thereof, called “crude fucoidan”. The last fraction of the supernatant from the different extract samples was subjected to ultrafiltration with membranes of different MWCO: 300, 100, 50, and 10 kDa in sequential manner. This process was performed by using an Amicon^®^ Stirred Cell unit (Millipore Corporation, Burlington, MA, USA). The optimal concentration of the samples was achieved by ultra-filtration of the samples through polyethersulfone membranes from Biomax™ (Merck Millipore, Darmstadt, Germany) using compressed air (5 bar) and magnetic stirring. After that, all samples were freeze-dried. The samples were freeze-dried in the L-300 Continuous Pro Modular, Buchi, Switzerland.

### 3.4. Measurement of Fucoidan Content

The crude fucoidan was measured for the fucoidan content of extracts obtained from the various samples using the method described by Dische & Shettles [83]. The fucose standards used had a concentration from 0.005 to 0.1 mg mL^−1^ and water was used as negative control. Then, 4.5 mL of 80% H_2_SO_4_ was added and incubated at 100 °C for 10 min. Then, 100 μL of 3% cysteine hydrochloride was added and kept at room temperature for 1 h. The absorbance at 396 and 430 nm was measured using a microplate reader (Epoch, BioTek, Winooski, VT, USA). The average fucose content in fucoidans was to be 50% and the result of the determination was multiplied by two, as described earlier [84].

### 3.5. Cell Culture

The rhesus monkey epithelial cell line MA104 was cultured in minimum essential medium (MEM) supplemented with 10% (*v*/*v*) foetal bovine serum (FBS), 1% (*v*/*v*) 2 mM L-glutamine, 1% (*v*/*v*) antibiotic solution (100 units mL^−1^ penicillin, 100 μg mL^−1^ streptomycin), and 0.25 μg mL^−1^ amphotericin B. Cells were maintained in 25 cm^2^ culture vessels at 37 °C in a Heraeus B5060 EK/CO_2_ thermostatic incubator with 95% of O_2_ and 5% CO_2_.

### 3.6. Algae Samples in the Vitro Assays

All algae extract powders assessed in the cytotoxicity and the antirotaviral activity assays were initially diluted 1:1 *w*/*v* in phosphate-buffered saline (PBS) composed of 0.14 M NaCl, 2.6 mM KCl, 8.1 mM Na_2_HPO_4_, 1 KH_2_PO_4_, 4 mM, and pH 7.4. For further dilutions, serum-free cell culture medium was used.

### 3.7. Cell Viability Assay

To evaluate the effect of algae samples on the viability of MA104 cells, a colorimetric assay based on the MTS reagent [84] was used. For this, the cells were cultured in 96-well plates with supplemented MEM under the conditions described above until confluence. To perform the assay, the culture medium was first removed from the wells, 200 μL/well of MEM without FBS were added, and the plate was incubated at 37 °C for 2 h. Next, the cell monolayer was washed twice with 200 μL/well of sterile PBS, and the plate was incubated for 2 h with 50 μL/well of the different algae solutions, of which the possible cytotoxicity was evaluated. After two washes with sterile PBS, 200 μL/well of MEM without FBS and 20 μL/well of MTS reagent were added, and the plate was incubated at 37 °C for 2 h. The absorbance was read at 490 nm using a Multiskan MS plate reader from Labsystem (Helsinki, Finland). The samples were analyzed with two replicates in two independent experiments (*n* = 4). The data were expressed as the average of the percentage of cell viability with respect to the control cells.

### 3.8. RV Propagation

The bovine WC3 strain (ATCC VR-2102) was propagated in the MA104 cell line, according to previously described procedures [85]. Briefly, confluent cell monolayers in 75 cm^2^ flasks were infected at a multiplicity of infection (MOI) of 0.1 with an aliquot of a RV suspension. Upon incubation at 37 °C for 1–3 days, the cytopathic effect was observed (cells fully lysed) and the RV was harvested by subjecting the vessel to three cycles of freezing (−80 °C) and thawing (room temperature). The resulting lysate, containing cells and virus, was clarified by centrifugation at 300× *g* for 15 min at 4 °C to remove cell debris. The supernatant, considered the infective viral suspension, was titrated, according to the procedures described previously [86], aliquoted in cryovials, and stored at −80 °C until use in the infectivity assays.

### 3.9. RV Neutralization Assay

MA104 cells were seeded in 96-well plates at a density of 1.8 × 10^4^ cells cm^−2^ and grown until 95% confluency. Cells were serum-starved for 2 h prior to the RV assay in MEM supplemented with 1% (*v*/*v*) 2 mM L-glutamine, 1% (*v*/*v*) antibiotic solution (100 units mL^−1^ penicillin, 100 μg mL^−1^ streptomycin), and 0.25 μg mL^−1^ amphotericin B. An aliquot of RV suspension was thawed and activated with trypsin (20 μg mL^−1^ final concentration) for 1 h at 37 °C. Next, the algae solutions were incubated with the RV suspensions in a 1:1 ratio in a 96-well conical bottom plate, for 1 h at 37 °C. Serum-free culture medium was used as negative control, and activated RV suspension diluted 1:1 with serum-free culture medium as positive control. The dilution of the virus suspension was adjusted with MEM to achieve a MOI of 0.02. The contents of the incubated plate were then transferred to the MA104 cell plate cells and incubated for 1 h at 37 °C. Afterwards, to facilitate the post-adsorption infection phase, 100 μL/well of MEM supplemented with 4% (*v*/*v*) FBS and 2 μg mL^−1^ trypsin were added to each well, and the plate was incubated at 37 °C for 16 h. Infected cells were afterwards detected by indirect immunofluorescence. Each sample was assessed in two replicates in four independent experiments (*n* = 8).

### 3.10. Cell Receptor Blocking Assay

The interaction of the algae with the cellular receptors of MA104 was studied to evaluate the influence that it may have on the antirotavirus activity. Similar to the neutralization assay, MA104 cells were seeded in 96-well plates and grown until confluency. Before the cell receptor blocking assay, the culture medium was removed, and the cells were incubated with serum-free medium for 2 h. Subsequently, 50 μL of the algae solutions were added in the plate containing the cells, which were incubated for 1 h at 4 °C to prevent the samples from penetrating the cells by endocytosis. Afterwards, wells were washed twice with 200 μL/well of PBS and cells were infected with the activated RV suspension, incubating them for 1 h at 37 °C. After washing the wells twice with 200 μL/well of PBS, 100 μL of MEM supplemented with 4% (*v*/*v*) FBS and 2 μg mL^−1^ trypsin were added to each well, and the plate was incubated at 37 °C for 16 h, subsequently proceeding to the determination of infection by indirect immunofluorescence. Each sample was assessed in duplicates in three independent experiments (*n* = 6).

### 3.11. Detection of RV Cell Infection by Indirect Immunofluorescence

After 16 h of the infection period, the cells were washed with 200 μL/well of sterile PBS. Cell fixation was then conducted by adding 300 μL/well of a solution of acetone: methanol: formaldehyde (1:1:1, *v*/*v*/*v*) and incubating for 3 min at 4 °C. Next, the plate was washed twice with sterile PBS and incubated with 100 μL/well of an antiserum against bovine RV UK strain obtained in lamb (diluted 1/300 in PBS with 4% (*v*/*v*) gelatin) at 37 °C for 2 h under gentle agitation. Then, the wells were washed three times with sterile PBS and incubated with 100 μL/well of FITC-conjugated donkey anti-sheep IgG antibody (diluted 1/200 in PBS with 4% (*v*/*v*) gelatin) for 1 h at 37 °C under gentle agitation. Finally, after washing three times with PBS, the fluorescent cells were counted in the Eclipse E400 fluorescence microscope with a Nikon FITC filter, and the Zen lite 2012 image processing software. The infectivity percentages were determined by enumerating fluorescent foci (infected cells) in each well and were expressed with respect to the infectivity obtained with the controls, which consisted of cells infected by the virus suspension without neutralizing agent, considered as 100%.

### 3.12. MIC Method

The MIC method was conducted following the procedure of Gupta et al. [87] with modifications. The seaweed Fraction 1 (F1) with a molecular weight >300 KDa obtained with HCl as extraction solvent was selected for its high antiviral activity and tested for antibacterial capacity against *C. jejuni*, *E. coli*, *S*. Typhimurium, and *L. monocytogenes*; *E. coli* K12. *E. coli*, and *S*. Typhimurium were cultured and incubated in TSA at 37 °C for 24 h; *L. monocytogenes* was incubated in TSA at 37 °C for 48 h; and *C. jejuni* was incubated at 42 °C for 48 h in MHA. After incubation, isolated colonies of all these bacteria were inoculated in 10 mL of MHB and incubated under the former specified conditions. From the obtained bacterial suspensions, different serial dilutions in MRD were performed to achieve a final concentration of 4 log CFU mL^−1^ in the wells. The extract was resuspended in sterile water to create a stock solution (25 mg mL^−1^), which was then serially diluted in MHB to obtain concentrations of 0.20, 0.39, 0.78, 1.56, 3.13, and 6.25 mg mL^−1^, which were added in a volume of 100 µL to the wells of a 96-well plate. Moreover, a volume of 100 µL of the assayed bacterium at a concentration of 4 log CFU mL^−1^ was added to the wells. Sample blanks were prepared using the extract alone and MHB. Additionally, a negative control was obtained using the bacterium treated with gentamicin (4 mg mL^−1^), while a positive control was obtained testing the bacterium alone. Microplates were covered with a plastic film and incubated during 24 h at 37 °C for *E. coli*, *S*. Typhimurium and 48 h for *L. monocytogenes*, and 48 h at 37 °C for *C. jejuni*. After incubation, the OD of the wells was measured at 600 nm using a Multiskan™ FC microplate photometer (ThermoFisher Scientific, Basingstoke, UK). The OD_600_ of the test well was subtracted from the OD_600_ of the blank and extract alone to minimize the interferences in absorbance caused by the medium and extract. Three independent experiments were conducted for each bacterial strain to ensure reproducibility.

### 3.13. Statistical Analysis

Statistical analysis was conducted utilizing the GraphPad Prism v8.0.2 software (GraphPad Software, San Diego, CA, USA) and results are presented as the mean ± standard deviation. The normality of the data was tested through the Shapiro–Wilk test. A two-tailed, unpaired student’s t-test was used for the comparison of means between the same neutralizing concentrations of extracts that followed normal distribution (whole extracts). In the case that normal distribution was not followed, the Mann–Whitney test was applied (crude fucoidan). Cell viability means and neutralization means obtained from the different MWCO fractions were compared applying one-way analysis of variance (ANOVA) followed by Tuckey’s multiple comparison test. Differences with *p* value ≤ 0.05 were considered statistically significant and confidence level was set at 95%.

## Figures and Tables

**Table 1:** Fucoidan content in various fractions from *F. vesiculosus* using different solvents.

Fraction	Extraction Solvent	Fucoidan Content
		(g fucoidan/100 g Extract)
Whole extract	HCl	25.69 ± 0.91
	CA	12.70 ± 0.10
Crude fucoidan	HCl	55.08 ± 0.89
	CA	47.30 ± 0.96

**Table 2:** Fucoidan content in various MWCO fractions from *F. vesiculosus* using different solvents.

Extraction Solvent	Fraction	Fucoidan Content
		(g fucoidan/100 g Extract)
HCl	F1 (>300 KDa)	63.59 ± 1.01
	F2 (<300 KDa)	3.78 ± 0.31
	F3 (<100 KDa)	2.16 ± 0.03
	F4 (<10 KDa)	2.18 ± 0.02
CA	F1 (>300 KDa)	42.74 ± 0.27
	F2 (<300 KDa)	1.12 ± 0.03
	F3 (<100 KDa)	0.73 ± 0.00
	F4 (<10 KDa)	0.73 ± 0.01

**Table 3:** MIC assay of the F1 (>300 KDa, HCl) fraction at different concentrations (mg mL^−1^) towards *E. coli, E. coli* K12, *S*. Typhimurium, *L*. *monocytogenes*, and *C. jejuni*.

Bacteria	Concentration (mg mL^−1^)					
	0.2	0.4	0.78	1.56	3.13	6.25
*E. coli*	+	+	+	+	+	-
*E. coli* K12	+	+	+	+	-	-
*S*. Typhimurium	+	-	-	-	-	-
*L. monocytogenes*	-	-	-	-	-	-
*C. jejuni*	-	-	-	-	-	-

## Data Availability

The data presented in this study are available on request from the corresponding author.
